# Aggregation of MAVS antiviral protein suggests a mechanism for increased type I interferon production in SLE

**DOI:** 10.1186/ar4635

**Published:** 2014-09-18

**Authors:** Wen-Hai Shao, Brendan A Hilliard, Daniel H Shu, Stephen O Priest, Philip L Cohen

**Affiliations:** 1 Temple University School of Medicine, Philadelphia, PA, USA

## Background

Patients with systemic lupus erythematosus (SLE) often have increased type I interferon levels (IFN-I) and activation of IFN-inducible genes (IFN signature). Because IFN-I has a key role in both the innate and adaptive immune responses, it is believed that heightened levels of this cytokine and the many genes it regulates may underlie the immune hyperreactivity and autoimmunity of SLE. The mechanism of IFN-I hyperproduction in SLE is under intense study. An important lead has emerged from our laboratory implicating the RIG-I antiviral pathway as a possible cause. In particular, the mitochondrial adaptor protein MAVS is a key intermediary in the RIG-I/MDA5 pathway, where viral RNA triggers a conformational change in RIG-I, leading to MAVS activation with subsequent IFN production. It has been reported that MAVS may form large prion-like aggregates, which might stimulate IFN-I production in a potent and prolonged fashion. We wondered whether such aggregates might be detectable *ex vivo *in SLE patients, and whether they might play a role in the sustained increased production of IFN-I.

## Methods

Peripheral blood mononuclear cells (PBMCs) were isolated from patients fulfilling ACR criteria for SLE, from healthy controls, and from patients with rheumatoid arthritis (RA). Mitochondrial lysates were prepared and MAVS aggregation was identified with a semi-denaturing agarose gel and confirmed by confocal immunofluorescent microscopy.

## Results

Twenty-two of 61 SLE patients showed clear MAVS aggregation, with essentially all of their MAVS protein in a high molecular weight aggregated form. None of the RA patients and only three of 33 healthy controls had abnormal MAVS. Clinical data analysis revealed that 82.4% MAVS-aggregate-positive SLE patients (mean age 46) had anti-SSA antibodies, compared with 40% MAVS-aggregate-negative patients (mean age 44), *P *< 0.01 by chi-square. 64.7% aggregation-positive patients had active SLE disease (skin rash, arthritis, increased ESR, low C4, and active renal disease), while only 10% of the aggregation-negative patients had active disease.

## Conclusions

Our findings are consistent with the notion that activation of the RIG-I pathway through inappropriate or persistent MAVS aggregation may lead to increased IFN-I production, immune stimulation, and systemic autoimmunity in SLE.

